# Homology Modeling and Molecular Docking Approaches for the Proposal of Novel Insecticides against the African Malaria Mosquito (*Anopheles gambiae*)

**DOI:** 10.3390/molecules27123846

**Published:** 2022-06-15

**Authors:** Luminita Crisan, Simona Funar-Timofei, Ana Borota

**Affiliations:** “Coriolan Dragulescu” Institute of Chemistry, 24 M. Viteazu Ave, 300223 Timisoara, Romania; lumi_crisan@acad-icht.tm.edu.ro (L.C.); timofei@acad-icht.tm.edu.ro (S.F.-T.)

**Keywords:** *Anopheles gambiae* mosquito, insecticide, in silico methods, toxicity predictions

## Abstract

Vector-borne infectious diseases are responsible for the deaths of over 700,000 people annually, than 400,000 of them resulting from malaria. The mosquito *Anopheles gambiae* is one of the dominant vector species of human malaria transmission. A significant issue of the conventional insecticides which target the arthropod borne infectious diseases is their induced resistance. To overcome this inconvenience, insecticides with new modes of action are required. One of the most promising targets for the development of new potential insecticides as evidenced by current studies is the D1-like dopamine receptor (DAR). To get a deeper understanding of the structural information of this receptor, the 3D homology model was built. The possible sites within the protein were identified and the most probable binding site was highlighted. The homology model along with a series of DAR antagonists with known activity against *Anopheles gambiae* larvae were used in docking experiments to gain insight into their intermolecular interactions. Furthermore, virtual screening of the natural compounds from the SPECS database led to the prediction of toxicity and environmental hazards for one potential new insecticide against the *Anopheles gambiae* mosquito.

## 1. Introduction

Globally, out of 700,000 annual deaths due to vector-borne infectious diseases, more than 400,000 of the result from more than 219 million malaria infections [1]. Responsible for malaria transmission are several *Anopheles* (*An.*) mosquito species. Among them, *An. gambiae* also known as the African malaria mosquito, is one of the most relevant, and has been most commonly reported (in 34 countries) [2]. According to the World Malaria Report of 2019 [3] the efforts to control the spread of malaria are hampered by the acquired resistance to many categories of insecticides, like pyrethroids, organochlorides [4], carbamates, and organophosphates [5], etc. Therefore, insecticides with new modes of action are an overall goal towards the eradication of malaria. as it appears also from the Innovative Vector Control Consortium (IVCC) calls [http://www.ivcc.com (accessed on 12 March 2020)]. In this regard, the G protein-coupled receptor (GPCR) family aroused great interest as a target for the development of next-generation insecticides [6]. Dopamine in invertebrates has a major role in many processes, such as development, locomotion, learning, courtship, etc. [7,8]. Some studies highlight that targeting the dopaminergic pathways may cause disrupted development in insects or even their death [8,9,10,11]. Dopamine receptors are members of class A GPCRs [12], that have seven transmembrane domain helices (generally, the site of the ligand-binding pocket) and additionally the eighth helix with a palmitoylated cysteine at the C terminal [13]. Dopamine receptors are split into two subfamilies: the D1-like family (D1 and D5 dopamine receptors) and the D2-like family (D2, D3, and D4 dopamine receptors), based on the coupling to either Gα_s,olf_ proteins or Gα_i/o_ proteins to stimulate or inhibit the production of the second messenger cAMP, respectively [12].

However, an interesting and unexpected finding was observed in the study conducted by Hill and collaborators [14], where D1-like dopamine receptor 2 of the *An. gambiae* species (AgDOP2) appeared to selectively couple to Gα_q_ signaling in vitro, as a potential divergence between the signaling mechanisms of invertebrate and mammalian DARs.

Several studies have pointed out the GPCR D1-like dopamine receptor (DAR) as a putative target for antagonist insecticides against Anopheles vectors [8,15].

It has been shown that the orthologous DAR of mosquitoes, Aedes aegypti (vector for yellow fever) and Culex quinquefasciatus (vector for West Nile virus) have in vitro a very similar pharmacological effect [11]. The D1-like dopamine receptor 2, namely DOP2 DAR, shows, on the one hand, high amino acid sequence identity (S.I.) among the vector insects like *An. gambiae*, *Ae. aegypti*, *C. quinquefasciatus*, *Ixodes scapularis*, (over 70% in transmembrane domains), but on the other hand lower S.I. to the human DARs, (under 55%) [11,15] These findings can be an asset to be considered in the rational design of new insecticides against the aforementioned vector insects. Taking into consideration the results of a recent study by Hill and collaborators [14] that attested to the in vitro and in vivo data for potential DAR antagonists against *An. gambiae*, we used in silico methods for predicting the possible intermolecular ligand-target interactions. The results obtained by the aforementioned group of researchers who cloned, molecular and pharmacologically characterized AgDOP2 (in Heck293 cells), as well as tested the in vivo activity of several antagonists against *An. gambiae* larvae represented the starting point for the current study. In the absence of the experimental structure of the target, homology modeling was involved in the building of the 3D structure. In the next step, the most probable location of the binding site was analyzed and assessed by several protocols and software and further utilized in molecular docking and virtual screening (VS) experiments. The insight gained through computational studies allowed us to understand the possibility of significant hydrophilic and hydrophobic interactions between the ligands and the amino acids of the DAR binding site, which may lead to the desired effect against the mosquito vector of malaria and the proposal of new potential insecticides. Furthermore, for the estimation of the selected compound environmental hazards, the bees’ toxicity, the oral acute rodent toxicity, and other toxicological endpoints were predicted.

## 2. Materials and Methods

### 2.1. Homology Modeling

A homology model, based on the AgDOP2 gene [GenBank ID: KU948225] (Supplementary Materials) of the *An. gambiae* species, corresponding to D1-like dopamine receptor 2 was obtained using the I-TASSER server [16] (https://zhanggroup.org/I-TASSER/ (accessed on 14 April 2020)). The I-TASSER server built the receptor 3D model using multiple threading templates. The top 10 templates (Supplementary Materials-Table S1) are identified by the threading program LOMETS [17], accessed by I-TASSER server, which uses different features in this regard, such as sequence identity, predicted secondary structure/solvent exposure, etc. Finally, the best models from the largest cluster of structures were selected by I-TASSER accessing the SPICKER program [18], which cluster the proteins based on their similarity.

The Protein Preparation Wizard from the Schrödinger package [19], and the Deep View Swiss-Pdb Viewer, version 3.7 [20] programs were used for refinement of the 3D structures (using standard settings), while the Procheck server [21] and Molprobity server (http://molprobity.biochem.duke.edu/ (accessed on 31 May 2022)) [22] were employed for the assessment and validation of the built homology model.

The quality of the raw homology model was first assessed by the Procheck server, and then Protein Preparation Wizard was involved in the energy minimization of the 3D structure using the OPLS 2005 force field, with a default setting of 0.3 Å for the root mean square deviation (RMSD). The 3D model was examined and the disordered regions with residues located in the disallowed areas of the Ramachandran plot followed a refining process with the aid of Refine Loops from Prime software [23]. When the structural issues persisted, a Swiss-PdbViewer Loop Database tool was used [20]. For a specific amino acid sequence (a loop) where problems have been identified, several loops were proposed from a database of known loops. For the selected loop, the following evaluation parameters: clash score, pair potential and force field were assessed. For situations where there was clash score, suitable rotamers were chosen for the residues involved in steric clashing to avoid steric hindrances. Swiss-PdbViewer was also engaged in energy minimization to acquire structures free of steric clashes [24]. Evaluation and refining steps followed iteratively until a good quality of the homology model was reached.

### 2.2. Site Identification

The SiteMap software [23], with default settings, was utilized to discover the possible sites within the 3D structure by using the scoring function (SiteScore) to recognize, evaluate and rank the regions that may be appropriate for ligand binding. SiteScore, the score utilized to determine and compare binding sites, is based on a weighted sum of properties like, size, enclosure, and hydrophilic terms [25]. The SiteScore function is presented in Equation (S1) (Supplementary Materials). Additionally, a Druggability Score (DScore) is computed. Dscore is composed of terms that promote ligand binding: suitable size, separation from solvent, and a term that corrects increasing hydrophilicity [25] as shown in Equation (S2) (Supplementary Materials).

### 2.3. Docking Protocol

Molecular docking experiments were performed using the fast rigid docking protocol of the FRED software from the OpenEye package [26,27,28,29,30,31]. The FRED program was found to be a very good choice in the reproduction of experimental poses and virtual screening experiments [30,31]. For docking, the multi-conformer structures of each ligand were generated usingOMEGA software [32,33], with default settings, and the receptor was prepared using the MakeReceptor software [34], starting from the homology model previously obtained. To assign a protonation state and find all possible tautomers of the compounds, they were further prepared with LigPrep software [35], using its default settings.

For each ligand, ten top-ranked docking poses were saved and visually analyzed using the Biovia Discovery Studio software [36]. Then, for the virtual screening experiment, the FRED program was employed as a docking engine for the 400 natural products from the SPECS database (http://www.specs.net, accessed on 26 February 2020), along with seven known inhibitors active against the D1-like dopamine receptor [14]. The widely used Chemgauss 4 (CG4) scoring function, which uses Gaussian functions to describe the shape and chemistry of molecules, implemented in FRED, was considered for a fast evaluation of protein-ligand interactions. FRED’s CG4 scoring functions account for the interactions which can include hydrogen bond interactions (H-acceptor and H-donors), shape, metal-chelator interactions, and desolvation effects for both protein and ligand. Compared with the CG3 scoring function, the hydrogen bonding and metal chelator terms have been improved in CG4 (the shape and implicit solvent interaction terms remain the same as in the old version). Therefore, the CG4 scoring function with better virtual screening and pose prediction performance was selected in our study.

### 2.4. Insecticide-Likeness Prediction

Quantitative estimate of the insecticide-likeness (QEI) [37] was calculated based on a function using six descriptors: molecular weight, logP, number of hydrogen bond acceptors, number of hydrogen bond donors, number of rotatable bonds and number of aromatic rings, which were computed with the aid of Instant JChem software [38].

### 2.5. Toxicity and Environmental Hazard Predictions

For the prediction of oral rodent acute and bee toxicities and other toxicological endpoints of the studied compounds, the ProTox-II [39,40], and BeeTox [41,42] tools were used, respectively. To estimate their potential bioaccumulation in aquatic species, the bioconcentration factor (BCF) was estimated using the EPI Suite program [43].

## 3. Results and Discussion

Since homology (comparative) modeling is recognized as the most precise in silico method to predict reliable 3D protein models from its amino acid sequences [44,45], in the absence of an experimentally determined structure, this method was applied using the I-TASSER server to build the D1-like dopamine receptor 2 of the *An. gambiae* species (DAR AgDOP2).

Once the raw homology models are obtained, they are further involved in a careful process of selection, refining, and optimization.

**Figure 1:**
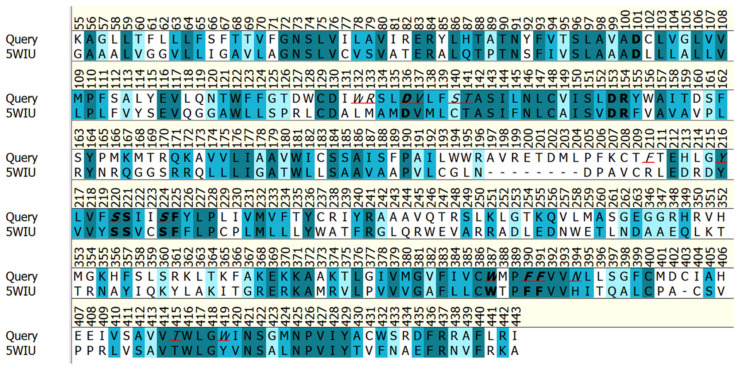
Sequence alignment of the query (GenBank ID: KU948225) and the best scored template (5WIUA), where the sequence identity and similarity are 30.6% and 54.1%, respectively. The conserved residues [14] are represented in bold, while the amino acids of the binding site, identified using the COFACTOR method and COACH meta-server, are italicized and underlined, respectively.

The confidence of each comparative model obtained by I-TASSER is quantitatively measured by the C-score that is typically in the range of [−5, 2], where a higher value of the C-score signifies a model with higher confidence, and vice versa. The best identified 3D model (out of five comparative models) obtained a C-score value of −2.4 and the sequence alignment of the query (GenBank ID: KU948225) and the best scored template (5WIUA) are presented in Figure 1. The “C-score” scoring function is calculated by taking into account the consensus significance score of the alignments of multiple threading templates and the convergence parameters of the structure assembly simulations [46]. The C-score equation is presented in Equation (S3) (Supplementary Materials). The PDB X-ray structure with the ID: 5WIU, determined at a good resolution of 1.96 Å, belongs to the human D4 Dopamine receptor in complex with nemonapride (a dopamine antagonist) [47].

**Figure 2:**
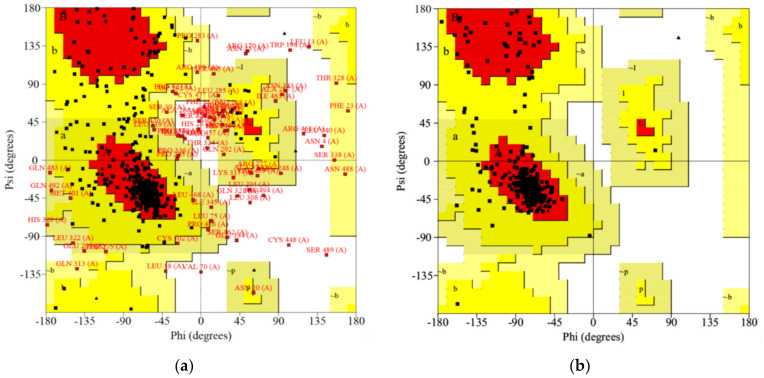
Ramachandran plot of DAR AgDOP2. (**a**) Raw model (with 61.4% residues located in most favored regions), and (**b**) Refined 3D homology model (with 90.2% residues in most favored regions).

The stereochemical quality of the 3D model was assessed using the PROCHECK server [21], mainly with the aid of the Ramachandran plot [48], which shows energetically allowed and forbidden zones for the Phi and Psi backbone torsional angles. They include disallowed (white areas), allowed (yellow areas), and most favored regions (red areas) regions (Figure 2). The residues that occur in the disallowed areas are associated with unfavorable conformations of the protein backbone where atoms are too close (more than the sum of their van der Waals radii).

The selected 3D model was examined and the disordered regions (Figure 2a) with many residues located in the disallowed zones (amino acid sequences: 1–54; 264–345; 444–495, Figure S1, Supplementary Material), especially those of long coil elements [49], were removed from the model after a previous check to ensure that they are not part of the key domains of the protein.

Starting from a raw structure with weak stereochemistry (Figure 2a), several iterative steps were taken to improve its quality. The Deep View Swiss-Pdb Viewer (version 3.7) [20], Prime [23], and Protein Preparation Wizard [19] tools were used for the refinement and optimization of the 3D structure of the protein. The homology model was examined and the following disordered regions: 66–74; 118–125; 157–162; 168–171; 40–405, with residues located in the disallowed areas of the Ramachandran plot were refined with the aid of Refine Loops from Prime. In addition, it was necessary to reshape certain regions of the protein (e.g., 78–82) using the database of known loops from the Swiss-PdbViewer Loop Database. To attest to a good quality of the homology model, over 90% of the amino acid residues should be in the most favored regions. As can be seen from Figure 2b, the refined model has 90.2% residues in the most favored regions and none in the banned areas. Additionally, the homology model quality was evaluated by PROCHECK assessing the following geometric and energetic parameters: main-chain parameters (Ramachandran plot quality assessment, peptide bond planarity, bad non-bonded interactions, Cα distortion, hydrogen bond energies, overall G-factor), side-chain parameters, and distorted geometry. All of these calculated parameters fit in the mean values, indicating a well refined and good quality homology model of the DAR AgDOP2 receptor (Figures S2 and S3, Supplementary Materials). In order to recognize the clash scores and outliers, the model assessment was also done with the Molprobity server (http://molprobity.biochem.duke.edu/ (accessed on 31 May 2022)) [22], obtaining a clash score for all atoms of 0.2 (99th percentile (N = 1784, all resolutions), where the 100th percentile is the best among the structures of comparable resolution and the 0th percentile is the worst. The clash score represents the number of serious steric overlaps (>0.4 Å) per 1000 atoms. The MolProbity score is 1.59 (93th percentile (N = 27,675, 0 Å–99 Å), where 100th percentile is the best among the structures of comparable resolution; 0th percentile is the worst. The MolProbity score combines the clash score, rotamer, and Ramachandran evaluations, normalized to be on the same scale as X-ray resolution. MolProbity identified one Ramachandran outlier—Thr68, which does not influence the protein binding site, given that it is outside the area of interest.

**Figure 3:**
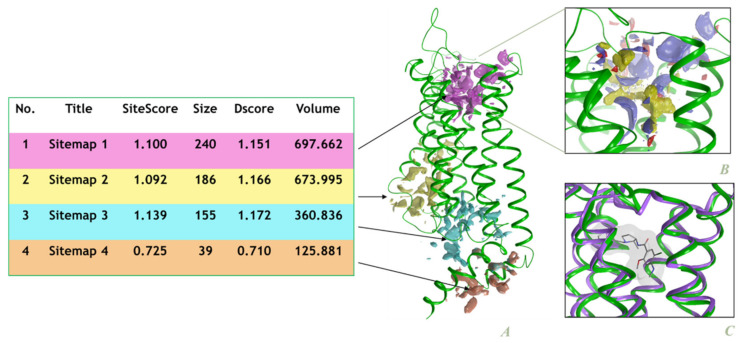
The parameters obtained using the SiteMap simulation; (**A**). The 3D structure of DAR AgDOP2; the four sitemaps are represented by colored surfaces; (**B**). In Sitemap1 the contour maps are depicted as transparent surfaces; hydrophobic map-yellow surface; hydrogen-bond donor map -blue surface; hydrogen-bond acceptor map-red surface; (**C**). The overlapping of the main sitemap (Sitemap1) of the homology model (the green ribbons) over the experimentally identified 5wiuA receptor (purple ribbons with the co-crystalized ligand).

In order to identify the possible binding sites within the protein, the SiteMap [23] top software was chosen, as it has been successfully used in previous studies [50,51]. The hydrophobic and hydrophilic contour site maps were generated. The hydrophilic maps were further divided into the donor, acceptor, and metal-binding regions. In the evaluation step, different properties corresponding to each site were estimated to complete the calculation. Four possible site maps were identified (Figure 3), and the main binding site found has a SiteScore of 1.100 and a volume of ~698 Å^3^, being located within the transmembrane bundle towards the extracellular side of the receptor. A superposition of the homology model over the top-scored template (PDB Id: 5WIU), is presented in Figure 3C, while the alignment of their amino acid sequences is shown in Figure 1. From Figure 3B,C, it can be observed that the best site identified using the SiteMap software (Sitemap 1) corresponds to the known binding site of the template crystal structure (5WIU). This hypothesis has been additionally confirmed using the COFACTOR method, which reasons based on the structure comparison and protein-protein networks [52], and also the COACH meta-server that combines multiple function annotation results [53] The same top-ranked binding site was achieved using both approaches, with a C-score of 0.29 and a cluster of 89 templates. The amino acids belonging to the aforementioned site can be seen in Figure 1.

Hill and coauthors have realized an interesting study on several potential antagonists (Table 1) against the DAR AgDOP2 vector to evaluate their insecticidal activity to larvae, expressed as IC_50_ values (µM) for the inhibition of dopamine-stimulated IP1 response in HEK-293 cell lines by DAR antagonists [14]. These compounds were further used in a molecular docking study in order to gain insight into intermolecular ligand-target interactions.

**Figure 4:**
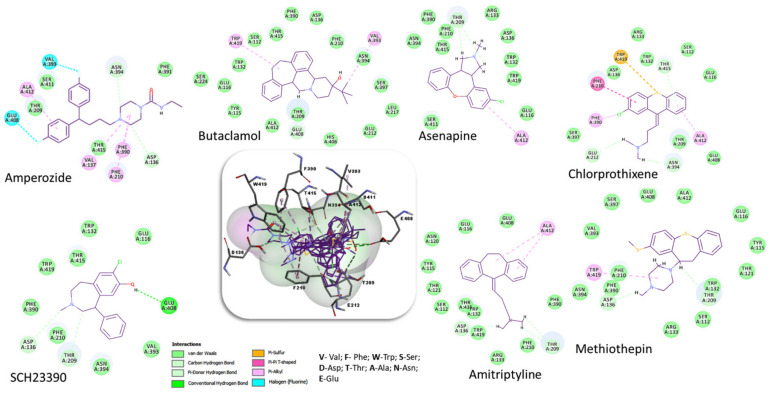
Superposition of the best poses identified for seven compounds (Table 1) into the DAR AgDOP2 binding site. The hydrogen bonds are depicted with green dashed lines, while hydrophobic interactions are presented with pink dashed lines.

The docking output files were generated with 10 poses for each compound. The best poses were chosen based on the Chemgauss4 score and key interactions. The interactions between the docked compounds and the amino acid residues of the binding site are presented in Table 1 and Figure 4.

**Figure 5:**
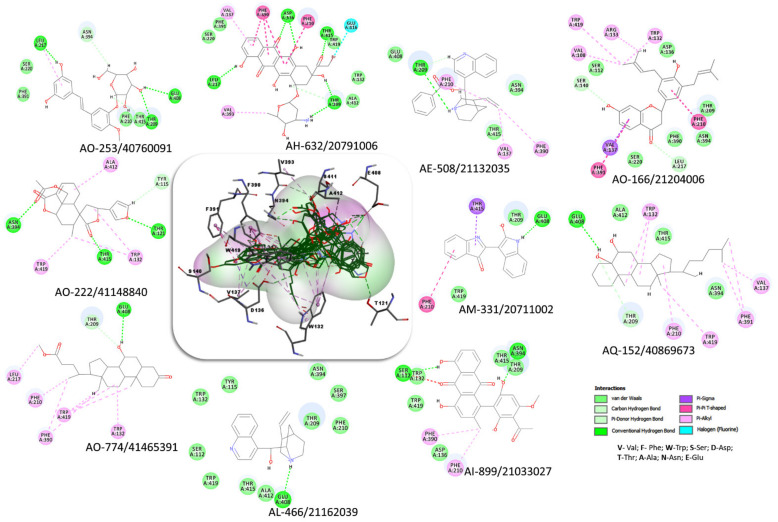
Superposition of the best poses identified for 10 compounds (Table 2) into the DAR AgDOP2 binding site. The hydrogen bonds are depicted with green dashed lines, while hydrophobic interactions are presented with pink dashed lines.

In order to identify new molecules with potential insecticidal activity and environmental safety that are most likely to bind to the DAR AgDOP2 target, vs. experiments were undertaken implying SPECS natural compounds repository. The ten top-ranked compounds (according to CG4 score) are presented in Table 2 and Figure 5.

The vs. results showed much better Chemgauss4 scores for the top compounds (Table 2) compared to those previously studied (Table 1). The evaluation of the interactions between the natural compounds and the amino acid residues revealed, as expected, mostly those conserved (D136, F390, F391and S140) and those predicted to belong to the binding site (N394, W132, V137, F210, W419, T415), as well as new ones (T209, E408, L217, S411, A412, V393, T121, E212). Among them, the most significant amino acid residues involved in hydrophilic interactions (hydrogen bonds) are D136, N394, E408, and T209, while the hydrophobic interactions are mainly realized with the following residues: F390, W419, A412, F210, and V137. By analyzing and comparing these results with the observed interactions between the 5WIU crystal structure (used as a template-Figure 1) and the nemonapride, the co-crystalized ligand (Figure 3C) it was observed that nemonapride interacts by hydrogen bonds with the conserved aspartate by D1153.32 (D136-Figure 1), and with the sidechain of S1965.42 (S220-Figure 1) [47].

Applying rule-based filters for predicting insecticide-like compounds, Tice et al. [54] found several criteria, such as molecular weight (MW between 150–500), logP (MLogP between 0–5), number of hydrogen bond donors (HBD ≤ 2), number of hydrogen bond acceptors (HBA between 1–8) and number of rotatable bonds (RB ≤ 9). The insecticide-likeness was estimated for the ten top-ranked compounds resulting from the vs. experiment using a desirability function based on the following six structural descriptors: molecular weight, logP, number of hydrogen bond acceptors, number of hydrogen bond donors, number of rotatable bonds, and number of aromatic rings [37]. The quantitative estimate of the insecticide-likeness (QEI) was calculated (Table 3). Compound **5** (teuscorodonin) was found with the highest desirability QEI score and fulfills the Tice criteria for insecticide-like compounds (the MlogP value, calculated with the Dragon software (Dragon Professional 5.5, 2007, Talete S.R.L., Milano, Italy) was of 3.84).

The ProTox-II server [39] was used to predict the toxic potential of the compounds taken in this study. ProTox-II makes predictions utilizing computer-based models trained on experimental data. Thus, involving similarity comparisons with known toxic chemicals, the acute toxicity class, the hepatotoxicity, and various toxicological endpoints (cytotoxicity, mutagenicity, carcinogenicity, and immunotoxicity), the toxicological pathways, and the toxicity targets were predicted for the targeted compounds based on trained machine learning models.

The evaluation of the toxicity prediction (Supplementary Material) indicates that among the top 10 compounds that resulted from VS, the vast majority belong to Toxicity Class IV and V, with the exception of no. 2 (Table 2), for which Class II has been assigned. The significance of the assignment by class can be seen in the caption of Table S2 (Supplementary Material). As can be seen in Tables S3 and S4 (Supplementary Material) two compounds (No. 3 and 10) pass all the evaluated toxicological criteria, while the rest of them having immunotoxicity issues.

The study of the impact of pesticides on insect pollinators, especially on bees, is a major and topical concern, therefore, a web server for the prediction of bee toxicity involving the Graph Convolutional Neural Network [42] method was used. Thus, the previously investigated molecules were further explored using the BeeTox tool [41] to predict their toxicity for the bees. The estimations showed non-toxic outcomes for the vast majority of compounds (except no. 3 and 9; Figures S4–S11, Supplementary Material). Compound **5** with the highest QEI estimated value and which passed the Tice insecticidal rules was found to be non-toxic for bees, according to this software (Figures S7 and S12, Supplementary Material).

One of the most significant parameters used for screening bioaccumulative and toxic substances is the bioconcentration factor (BCF). BCF is an essential notion in environmental risk assessment referring to the ability of a contaminant to be taken up by organisms from the water [55]. The computation of BCFs has been achieved with the aid of the BCFBAF Program from EPI Suite [43]. The results of the estimation showed low bioconcentration potential for aquatic species (BCF < 1000, https://www.epa.gov/sites/default/files/2015-05/documents/05.pdf, accessed on 10 January 2022) for all the ten natural investigated compounds (Supplementary Material-Table S5).

An analysis of the vs. results indicated that some natural products identified in the present paper as potential insecticides against the *An. gambiae* species were previously shown to function as repellents against various species. Thus, acylated rhaponticin (a derivative of compound ranked no. 1 in Table 2), isolated from eucalyptus rubida, was found as a repellent against the blue mussel mytilus edulis, ref. [56] while cinchonine (Table 2) was found to be a potent insecticide against Mythimna separata (Walker) in a recent study [57]. Cinchonine as an alkaloid found in the Cinchona tree along with other analogous (quinine, quinidine) [58] is also effective against Plasmodium falciparum, the predominant species that causes malaria [59]. As it is known, alkaloids belong to a class of compounds with a wide range of biological activities, including activity against the malaria vector *Anopheles gambiae* [60].

## 4. Conclusions

In the absence of an experimentally determined structure, comparative modeling is considered to be the most reliable in silico method to predict 3D protein models from their amino acid sequences. Thushomology modeling was applied using the I-TASSER server to build the DAR DOP2 receptor of *An. gambiae* species. An unsatisfactory percentage of residues (61.4%) in the most favored regions of Ramachandran plot was observed in the raw homology model. The homology model has undergone an intensive process of refinement until a good percentage of 90.2% residues in the most favored regions was obtained.

With the aid of the SiteMap software, four sites were identified; the primary site was, also, discovered by the COFACTOR method and COACH meta-server and was confirmed by the superposition over the experimental structure of human Dopamine D4 receptor (PDB ID: 5wiuA).

The homology model obtained was involved in a virtual screening process in order to discover new and safe potential insecticides targeting the DAR DOP2 receptor of *An. gambiae*, a dominant vector species of human malaria transmission. The top 10 natural compounds ranked were further investigated in terms of ligand-receptor interactions, and their insecticidal and toxicological profile was predicted. The results of the study predicted that the investigated compound 5 (teuscorodonin) might have potential insecticidal characteristics against *An. gambiae* and is not bioaccumulative for aquatic species. The predicted toxicities indicate that the known natural compound teuscorodonin belongs to the toxicity Class IV, being inactive for the following endpoints: mutagenicity, hepatotoxicity, and cytotoxicity, and is not toxic for bees. Furthermore, this study, by highlighting ligand-binding site interactions, can facilitate the first step to a better understanding of a potential mode of action of the DAR antagonists against AgDOP2 receptors.

## Figures and Tables

**Table 1:** DAR AgDOP2 antagonists along with their IC_50_ values and the docking results.

Compound	2D Structure	pIC_50_^1^	CG4 Score	H Bonds ^2^	Hydrophobic Interactions ^3^
Amitriptyline	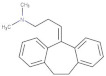	6.638	−8.522	D136, T209	A412
Amperozide	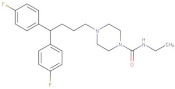	4.726	−8.901	D136, N394,	F390, F210, A412
Asenapine	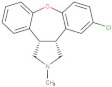	9.155	−8.935	D136, T209	A412
Butaclamol	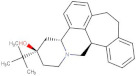	5.921	−8.947	E408	V393, W419
Chlorprothixene	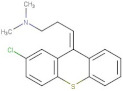	6.398	−8.056	N394, E212, T415	F390, F210, A412
Methiothepin	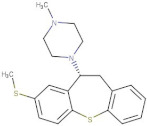	6.854	−7.051	D136, T209	S411, W419
SCH23390	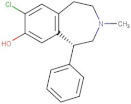	5.444	−8.706	D136, E408, T209	

^1^ The insecticidal activity values are taken from the reference [14] and molar converted to pIC_50_ values; ^2^ Amino acid residues involved in hydrogen bond formation; ^3^ Amino acid residues involved in hydrophobic interactions.

**Table 2:** The top-ranked compounds from the Specs database selected through vs. experiment.

No.	Specs ID Number	2D Structure	Name	CG4 Score	H Bonds	Hydrophobic Interactions
1	AO-253/40760091	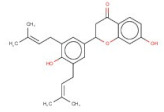	Rhaponticin (Rhapontin)	−14.354	N394 T209 E408 S411 L217	L217
2	AH-632/20791006	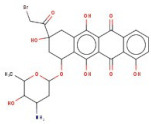	3-(bromoacetyl)-3,5,10,12-tetrahydroxy-6,11-dioxo-1,2,3,4,6,11-hexahydro-1-naphthacenyl 3-amino-2,3,6-trideoxyhexopyranoside	−12.776	D136 T209 T415 L217	F390 V393 V137 F210
3	AE-508/21132035	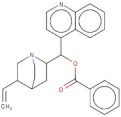	O-Benzoylcinchonine	−12.212	T209	F390 V137 F210
4	AO-166/21204006	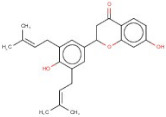	Abysinnone	−11.947	S140 L217	F391 V137 W132 W419 F210 R133 V108
5	AO-222/41148840	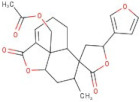	Teuscorodonin	−11.807	T121 N394 T415 Y115	W132 W419 A412
6	AM-331/20711002	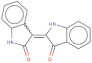	Indirubin	−11.676	E408	T415 F210
7	AI-899/21033027	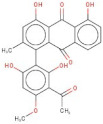	1-(3-acetyl-2,6-dihydroxy-4-methoxyphenyl)-4,5-dihydroxy-2-methylanthra-9,10-quinone	−11.546	N394 S112	F390 F210
8	AQ-152/40869673	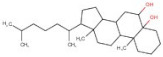	Cholestane-3,5,6-triol	−11.445	E408	F391 W132 V137 W419 F210
9	AO-774/41465391	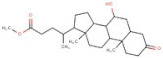	3,7-Dihydroxycholan-24-oic acid	−11.336	E408 T209	F390 W132 F210 W419 L217
10	AL-466/21162039	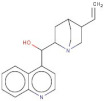	Cinchonine	−11.277	E408	-

**Table 3:** Structural descriptors based on which the insecticide-likeness (QEI) was calculated.

No.	MW	LogP	HBA	HBD	RB	arR	QEI
1	420.41	0.98	9	6	6	2	0.124
2	593.40	2.45	10	6	4	2	0.092
3	399.51	5.17	2	1	6	3	0.543
4	392.50	5.95	4	2	5	2	0.441
5	400.43	2.53	3	0	4	1	0.751
6	261.26	3.32	3	1	1	3	0.442
7	433.39	5.22	8	3	2	3	0.159
8	404.68	6.67	2	2	5	0	0.540
9	404.59	4.07	3	1	5	0	0.686
10	295.41	2.67	2	2	3	2	0.525

## Data Availability

The data presented in this study are available on request from the corresponding author.

## Supplementary Materials

The following supporting information can be downloaded at: https://www.mdpi.com/article/10.3390/molecules27123846/s1, Table S1: Top 10 threading templates used by I-TASSER; Figure S1: Predicted normalized B-factor; Table S2: Physicochemical parameters and oral toxicity prediction results for the selected NPs and D1-like DAR antagonists; Table S3: Toxicity Model Report—Prediction of various toxicity endpoints; Table S4: Toxicological pathways prediction; Figure S2: Main-chain parameters of DAR AgDOP2; Figure S3: Side-chain parameters of DAR AgDOP2; Figure S4: AO-253/40760091 (Compound **1** from Table 2); Figure S5: AH-632/20791006 (Compound **2** from Table 2); Figure S6: AO-166/21204006 (Compound **4** from Table 2); Figure S7: AO-222/41148840 (Compound **5** from Table 2); Figure S8: AM-331/20711002 (Compound **6** from Table 2); Figure S9: AI-899/21033027 (Compound **7** from Table 2); Figure S10: AQ-152/40869673 (Compound **8** from Table 2); Figure S11: AL-466/21162039 (Compound **10** from Table 2); Table S5: Bioconcentration Factors (BCFs) computed with BCFBAF Program from EPI Suite; Figure S12: The compound **5** into the DAR AgDOP2 binding site.
