# Supplementary material for: Homology Modeling and Molecular Docking Approaches for the Proposal of Novel Insecticides against the African Malaria Mosquito (Anopheles gambiae)
Source: Molecules. 2022 Jun 15;27(12):3846. doi: 10.3390/molecules27123846 (PMC9227062; doi:10.3390/molecules27123846)
Supplement: Supplementary file 1 [file molecules-27-03846-s001.zip › molecules-1745564-Supplementary Materials.pdf]

molecules

MDPI

Supplementary Materials

Homology Modeling and Molecular Docking Approaches for the Proposal of Novel Insecticides against the African Malaria Mosquito (*Anopheles gambiae*)

Luminita Crisan †, Simona Funar-Timofei † and Ana Borota *

"Coriolan Dragulescu" Institute of Chemistry, 24 M. Viteazu Ave, 300223 Timisoara, Romania; lumi_crisan@acad-icht.tm.edu.ro (L.C.), timofei@acad-icht.tm.edu.ro (S.F.-T.)

* Correspondence: ana_borota@acad-icht.tm.edu.ro

† These authors contributed equally to the work.

Protein Sequence of the query AgDOP2 DAR (GenBank: KU948225.1):

"MMSNASEVPWEFLQLTALTNISFYNVSHGSVEFSINCGSVVPNFSNYIVSLPNDKAGL
LTFLLLFSFTTVFGNSLVILAVIRERYLHTATNYFVTSLAVADCLVGLVVMPFSALYEVL
QNTWFFGTDWCDIWRSLDVLFSTASILNLCVISLDRYWAITDSFSYPMKMTRQKAVVL
IAAVWICSSAISFPAILWWRAVRETDMLPFKCTFTEHLGYLVFSSIISFYLPLIVMVFTYC
RIYRAAAVQTRSLKLGTKQVLMASGELQLTLRIHRGGTTRERLNHPRLAASDHYQQF
QHLDQMQPNTQPSLSTQHQFPLKKLHQLQTTNSTPDDPDEEPLSALENNGIGGRHR
VHMGKHFSLSRKLTKFAKEKKAAKTLGIVMGVFIVCWMPFFVVNLLSGFCMDCIAHE
EIVSAVVTWLGWINSGMNPVIYACWSRDFRRAFLRILCVCCPRKLRLKYQPTRRSIAS
QRLASRRCYSTCSLHGIQQVRQNSCEQTHI"

$$SiteScore = 0.0733\, n^{1\backslash 2} + 0.6688\, e - 0.20\, p \text{ (eq. S1)}$$

where "n is the number of site points (up to 100), e is the enclosure score, and p is the hydrophilic score, capped at 1.0 (the average for the submicromolar sites) to limit the impact of hydrophilicity in charged and highly polar sites." [26]

$$Dscore = 0.094\, n^{1\backslash 2} + 0.60\, e - 0.324\, p \text{ (eq. S2)}$$

where "n is the number of site points found for the site, capped at 100, e is the degree of enclosure of the site, and p is the hydrophilic score computed for the site". [26]

$$C - score = \ln\left(\frac{M}{M_{tot}} \cdot \frac{1}{\langle RMSD \rangle} \cdot \frac{\prod_{i=1}^{4} Z(i)}{\prod_{i=1}^{4} Z_0(i)}\right) \text{ (eq. S3)}$$

where M is the multiplicity of structures in the SPICKER cluster; M_{tot} is the total number of the I-TASSER structure decoys used in the clustering; ⟨RMSD⟩ is the average RMSD of the decoys to the cluster centroid; Z(i) is the highest Z-score (the energy to mean in the unit of standard deviation) of the templates by the i th PPA threading program and $Z_0(i)$ is a program-specified Z-score cutoff for distinguishing between good and bad templates, i.e. $Z_0(1) = 7.0$, $Z_0(2) = 8.5$, $Z_0(3) = 8.0$, $Z_0(4) = 10.5$" [47].

Table S1. Top 10 threading templates used by I-TASSER.

Rank	PDB Hit	Iden1	Iden2	Cov	Norm. Z-score
1	5wiuA	0.26	0.24	0.75	2.32
2	2rh1A	0.33	0.31	0.79	4.21
3	5wiuA	0.26	0.24	0.75	3.16
4	4ib4	0.30	0.25	0.75	1.38
5	3uon	0.25	0.25	0.79	1.08
6	5wiuA	0.27	0.24	0.75	3.21
7	4ib4	0.29	0.25	0.73	1.53
8	2rh1A	0.32	0.31	0.79	4.06
9	5wiuA	0.28	0.24	0.75	2.49
10	6drxA	0.28	0.26	0.74	3.94

1. Rank of templates represents the top ten threading templates used by I-TASSER.
2. Ident1 is the percentage sequence identity of the templates in the threading aligned region with the query sequence.
3. Ident2 is the percentage sequence identity of the whole template chains with query sequence.
4. Cov represents the coverage of the threading alignment and is equal to the number of aligned residues divided by the length of query protein.
5. Norm. Z-score is the normalized Z-score of the threading alignments. Alignment with a Normalized Z-score>1 mean a good alignment and vice versa.

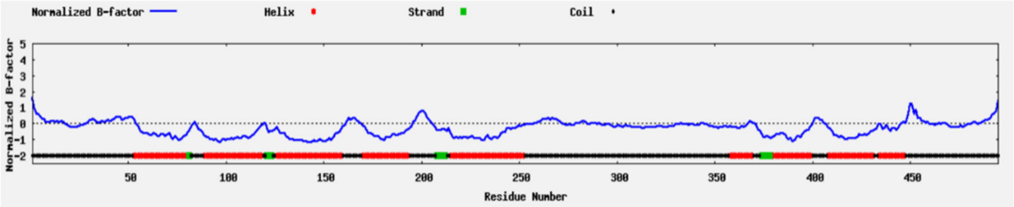

Figure S1. Predicted normalized B-factor.

The physicochemical parameters and oral toxicity prediction were calculated with online variant of ProTox II [https://tox-new.charite.de/protox_II/index.php?site=home – accessed on august 02, 2021] and presented in Tables S2-S4.

Table S2. Physicochemical parameters, predicted toxicity class and oral acute rodent toxicity prediction results for the selected NPs and D1-like DAR antagonists.

No.	SPECS ID / No.	MR	TPSA	logP	$LD_{50\ pred}$	Class
1	AO-253/40760091	106.5	149.07	0.46	4000	**V**
2	AH-632/20791006	134.9	196.84	1.8	7	**II**
3	AE-508/21132035	122.88	42.43	4.97	600	**IV**
4	AO-166/21204006	116.99	66.76	5.82	2000	**IV**
5	AO-222/41148840	99.8	92.04	3.11	555	**IV**
6	AM-331/20711002	81.89	58.2	2.93	1000	**IV**
7	AI-899/21033027	113.04	144.19	3.91	2100	**V**
8	AQ-152/40869673	125.29	40.46	6.58	2000	**IV**
9	AO-774/41465391	115.96	63.6	4.77	3881	**V**
10	AL-466/21162039	94.21	37.56	3.32	720	**IV**
	Amitriptyline	90.96	3.24	4.17	99	**III**
	Amperozide	118.78	35.58	4.49	500	**IV**
	Asenapine	84.90	12.47	4.20	244	**III**
	Butaclamol	115.47	23.47	4.78	350	**IV**
	Chlorprothixene	92.28	28.54	5.19	50	**III**
	Methiothepin	112.09	57.08	4.28	68	**III**
	SCH23390	87.01	23.47	3.60	185	**III**

MR- Molecular refractivity; TPSA- Topological Polar Surface Area; logP - Octanol/water partition coefficient(logP); LD50 pred - Predicted LD50 (mg/kg); Class - Predicted Toxicity Class; Toxic doses are often given as LD50 values in mg/kg body weight. The LD50 is the median lethal dose meaning the dose at which 50% of test subjects die upon exposure to a compound; Class I - fatal if swallowed ($LD_{50} \leq 5$); Class II - fatal if swallowed ($5 < LD_{50} \leq 50$); Class III - toxic if swallowed ($50 < LD_{50} \leq 300$); Class IV- harmful if swallowed ($300 < LD_{50} \leq 2000$); Class V- may be harmful if swallowed ($2000 < LD_{50} \leq 5000$); Class VI - non-toxic ($LD_{50} > 5000$).

Table S3. Toxicity Model Report - Prediction of various toxicity endpoints*.

No.	SPECS ID		Organ toxicity	Toxicity endpoints			
			Hepatotoxicity	Carcinogenicity	Immunotoxicity	Mutagenicity	Cytotoxicity
**1**	**AO-253/40760091**	**Prediction**	I	I	A	I	I
1	AO-253/40760091	**Probability**	0.81	0.82	0.96	0.75	0.79
2	AH-632/20791006	**Prediction**	I	I	A	A	A
2	AH-632/20791006	**Probability**	0.79	0.7	0.99	0.87	0.57
3	AE-508/21132035	**Prediction**	I	I	I	I	I
3	AE-508/21132035	**Probability**	0.94	0.51	0.82	0.75	0.79
4	AO-166/21204006	**Prediction**	I	I	A	I	I
4	AO-166/21204006	**Probability**	0.69	0.65	0.88	0.67	0.83
5	AO-222/41148840	**Prediction**	I	A	A	I	I
5	AO-222/41148840	**Probability**	0.87	0.54	0.99	0.83	0.82
6	AM-331/20711002	**Prediction**	I	A	I	A	I
6	AM-331/20711002	**Probability**	0.5	0.59	0.89	0.58	0.64
7	AI-899/21033027	**Prediction**	I	I	A	I	I
7	AI-899/21033027	**Probability**	0.73	0.63	0.99	0.75	0.84
8	AQ-152/40869673	**Prediction**	I	I	A	I	I
8	AQ-152/40869673	**Probability**	0.79	0.64	0.97	0.6	0.88
9	AO-774/41465391	**Prediction**	I	I	A	I	I
9	AO-774/41465391	**Probability**	0.62	0.71	0.9	0.94	0.72
10	AL-466/21162039	**Prediction**	I	I	I	I	I
10	AL-466/21162039	**Probability**	0.87	0.69	0.61	0.63	0.77
	Amitriptyline	**Prediction**	I	I	I	I	I
	Amitriptyline	**Probability**	0.55	0.73	0.58	0.79	0.63
	Amperozide	**Prediction**	I	I	I	I	I
	Amperozide	**Probability**	0.89	0.7	0.93	0.74	0.88
	Asenapine	**Prediction**	I	I	I	I	I
	Asenapine	**Probability**	0.66	0.58	0.93	0.53	0.59
	Chlorprothixene	**Prediction**	A	I	A	I	I
	Chlorprothixene	**Probability**	0.6	0.74	0.94	0.71	0.63

Methiothepin	**Prediction**	I	I	I	I	I
Methiothepin	**Probability**	0.63	0.85	0.59	0.64	0.68
SCH23390	**Prediction**	I	I	I	I	I
SCH23390	**Probability**	0.78	0.59	0.66	0.64	0.5
Butaclamol	**Prediction**	I	I	I	I	I
Butaclamol	**Probability**	0.94	0.66	0.93	0.67	0.59

*I- Inactive; A- Active.

Table S4. Toxicological pathways prediction*.

**No.**	**SPECS ID**		**Tox21-Nuclear receptor signalling pathways**											
			**AhR**	**AR**	**AR-LBD**	**Aromatase**	**ER**	**ER-LBD**	**PPAR-Gamma**	**nrf2/ARE**	**HSE**	**MMP**	**p53**	**ATAD5**
1	AO-253/40760091	**Prediction**	I	I	I	I	I	I	I	I	I	I	I	I
1	AO-253/40760091	**Probability**	0.76	0.95	0.98	0.96	0.79	0.95	0.97	0.95	0.95	0.86	0.75	0.94
2	AH-632/20791006	**Prediction**	I	I	A	A	I	I	I	I	I	A	A	I
2	AH-632/20791006	**Probability**	0.89	0.98	0.5	0.54	0.67	0.63	0.91	0.95	0.95	0.58	0.58	0.54
3	AE-508/21132035	**Prediction**	I	I	I	I	I	I	I	I	I	I	I	I
3	AE-508/21132035	**Probability**	0.52	0.95	0.89	0.95	0.83	0.96	0.96	0.85	0.85	0.94	0.94	0.94
4	AO-166/21204006	**Prediction**	I	I	I	I	I	I	I	I	I	A	I	I
4	AO-166/21204006	**Probability**	0.59	0.96	0.95	0.7	0.62	0.8	0.76	0.77	0.77	0.69	0.54	0.86
5	AO-222/41148840	**Prediction**	I	A	A	I	A	I	I	I	I	I	I	I
5	AO-222/41148840	**Probability**	0.95	0.8	0.53	0.5	0.54	0.95	0.87	0.94	0.94	0.58	0.81	0.97

6	AM-331/20711002	**Prediction**	I	I	I	I	I	I	I	I	I	A	I	I
6	AM-331/20711002	**Probability**	0.5	0.93	0.98	0.89	0.86	0.96	0.97	0.88	0.88	0.61	0.74	0.82
7	AI-899/21033027	**Prediction**	A	I	I	I	A	I	I	I	I	A	I	A
7	AI-899/21033027	**Probability**	0.53	0.98	0.99	0.82	0.52	0.66	0.94	0.87	0.87	0.85	0.54	0.52
8	AQ-152/40869673	**Prediction**	I	I	I	I	I	I	I	I	I	I	I	I
8	AQ-152/40869673	**Probability**	0.99	0.95	0.94	0.99	0.59	0.75	0.99	0.91	0.91	0.54	0.94	0.97
9	AO-774/41465391	**Prediction**	I	I	I	I	I	I	I	I	I	I	I	I
9	AO-774/41465391	**Probability**	0.98	0.82	0.82	0.98	0.8	0.77	0.99	0.99	0.99	0.76	0.93	0.96
10	AL-466/21162039	**Prediction**	I	I	I	I	I	I	I	I	I	I	I	I
10	AL-466/21162039	**Probability**	0.53	0.97	0.98	0.89	0.85	0.98	0.99	0.96	0.96	0.91	0.95	0.98
	Amitriptyline	**Prediction**	I	I	I	I	I	I	I	I	I	I	I	I
	Amitriptyline	**Probability**	0.97	0.99	0.99	0.91	0.93	0.96	0.98	0.98	0.98	0.78	0.94	0.99
	Amperozide	**Prediction**	I	I	I	I	I	I	I	I	I	I	I	I
	Amperozide	**Probability**	0.93	0.98	0.97	0.93	0.89	0.99	0.98	0.98	0.98	0.89	0.96	0.99
	Asenapine	**Prediction**	I	I	I	I	I	I	I	I	I	I	I	I
	Asenapine	**Probability**	0.77	0.99	0.96	0.8	0.74	0.88	0.97	0.93	0.93	0.84	0.87	0.96

Chlorprothixene	**Prediction**	I	I	I	I	I	I	I	I	I	I	I	I
Chlorprothixene	**Probability**	0.98	0.99	1	0.96	0.98	0.99	0.99	0.99	0.99	0.76	0.79	0.99
Methiothepin	**Prediction**	I	I	I	I	I	I	I	I	I	I	I	I
Methiothepin	**Probability**	0.97	0.99	0.99	0.97	0.98	0.99	0.97	0.98	0.98	0.94	0.98	0.99
SCH23390	**Prediction**	I	I	I	I	I	I	I	I	I	I	I	I
SCH23390	**Probability**	0.89	0.97	0.98	0.87	0.76	0.94	0.98	0.97	0.97	0.85	0.93	0.99
Butaclamol	**Prediction**	I	I	I	I	I	I	I	I	I	I	I	I
Butaclamol	**Probability**	0.88	0.94	0.98	0.91	0.88	0.98	0.99	0.97	0.97	0.77	0.9	0.98

*I- Inactive; A- Active; AhR - Aryl hydrocarbon Receptor (AhR); AR- Androgen Receptor (AR); AR-LBD - Androgen Receptor Ligand Binding Domain (AR-LBD) ER- Estrogen Receptor Alpha (ER); ER-LBD - Estrogen Receptor Ligand Binding Domain (ER-LBD); PPAR-Gamma - Peroxisome Proliferator Activated Receptor Gamma (PPAR-Gamma); nrf2/ARE - Nuclear factor (erythroid-derived 2)-like 2/antioxidant responsive element (nrf2/ARE); HSE- Heat shock factor response element (HSE); MMS - Mitochondrial Membrane Potential (MMP); p53 - Phosphoprotein (Tumor Supressor) p53; ATAD5- ATPase family AAA domain-containing protein 5 (ATAD5).

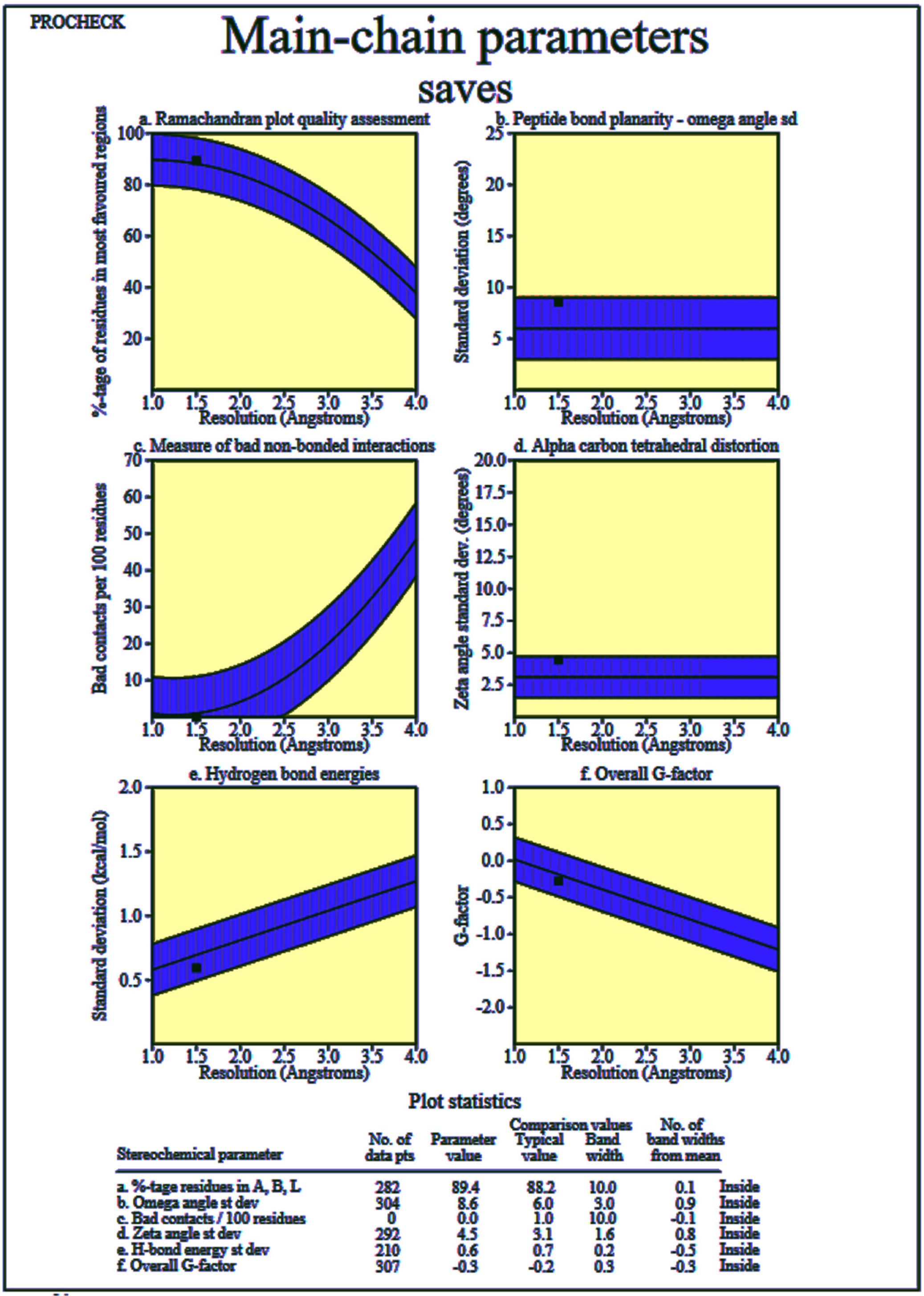

Stereochemical parameter	No. of data pts	Parameter value	Comparison values Typical value	Band width	No. of band widths from mean	
a. %-tage residues in A, B, L	282	89.4	88.2	10.0	0.1	Inside
b. Omega angle st dev	304	8.6	6.0	3.0	0.9	Inside
c. Bad contacts / 100 residues	0	0.0	1.0	10.0	-0.1	Inside
d. Zeta angle st dev	292	4.5	3.1	1.6	0.8	Inside
e. H-bond energy st dev	210	0.6	0.7	0.2	-0.5	Inside
f. Overall G-factor	307	-0.3	-0.2	0.3	-0.3	Inside

Figure S2. Main-chain parameters of DAR AgDOP2.

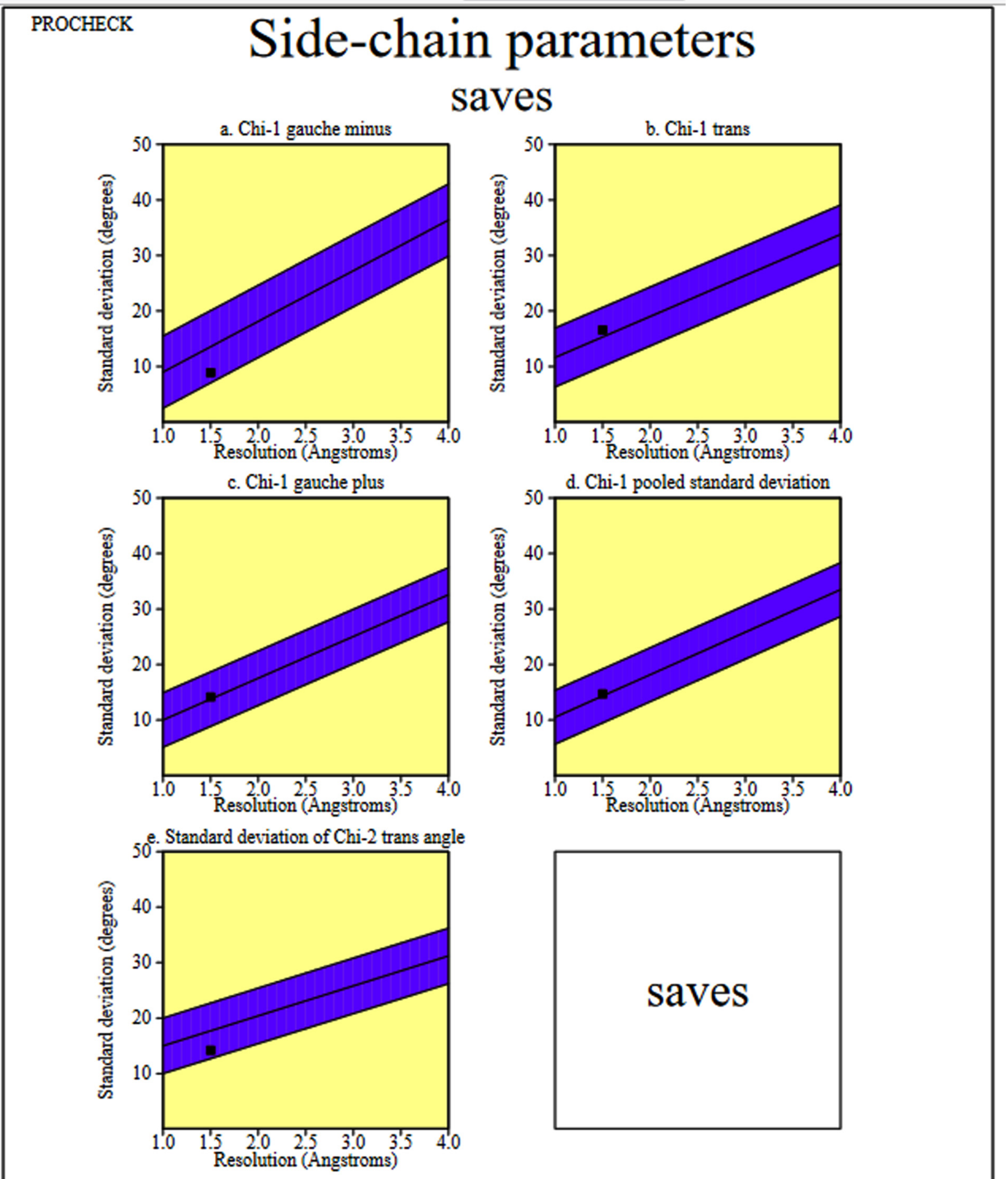

Plot statistics

Stereochemical parameter	No. of data pts	Parameter value	Comparison values Typical value	Band width	No. of band widths from mean	
a. Chi-1 gauche minus st dev	41	8.9	13.6	6.5	-0.7	Inside
b. Chi-1 trans st dev	112	16.6	15.3	5.3	0.2	Inside
c. Chi-1 gauche plus st dev	107	14.2	13.8	4.9	0.1	Inside
d. Chi-1 pooled st dev	260	14.7	14.3	4.8	0.1	Inside
e. Chi-2 trans st dev	57	14.2	17.7	5.0	-0.7	Inside

Figure S3. Side-chain parameters of DAR AgDOP2.

The bee toxicity prediction , was calculated using the BeeTox tool [http://chemyang.ccnu.edu.cn/ccb/server/beetox/index.php/prediction/index - accessed on March 2022] and are presented in Figures S4-S11.

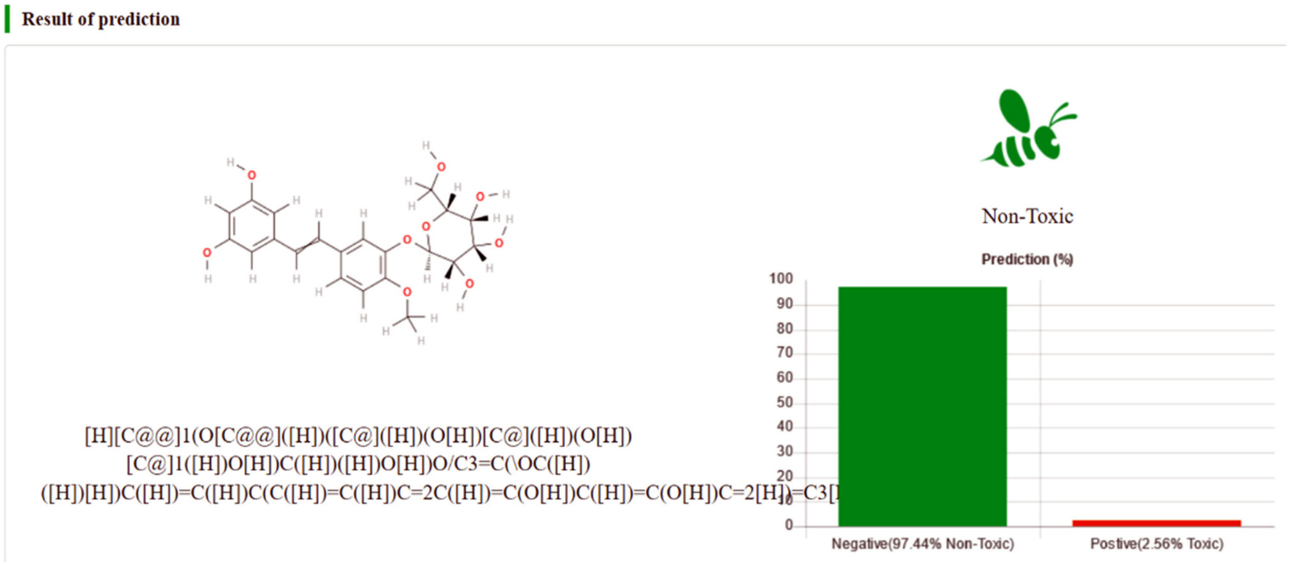

Figure S4. AO-253/40760091 (Compound 1 from Table 2).

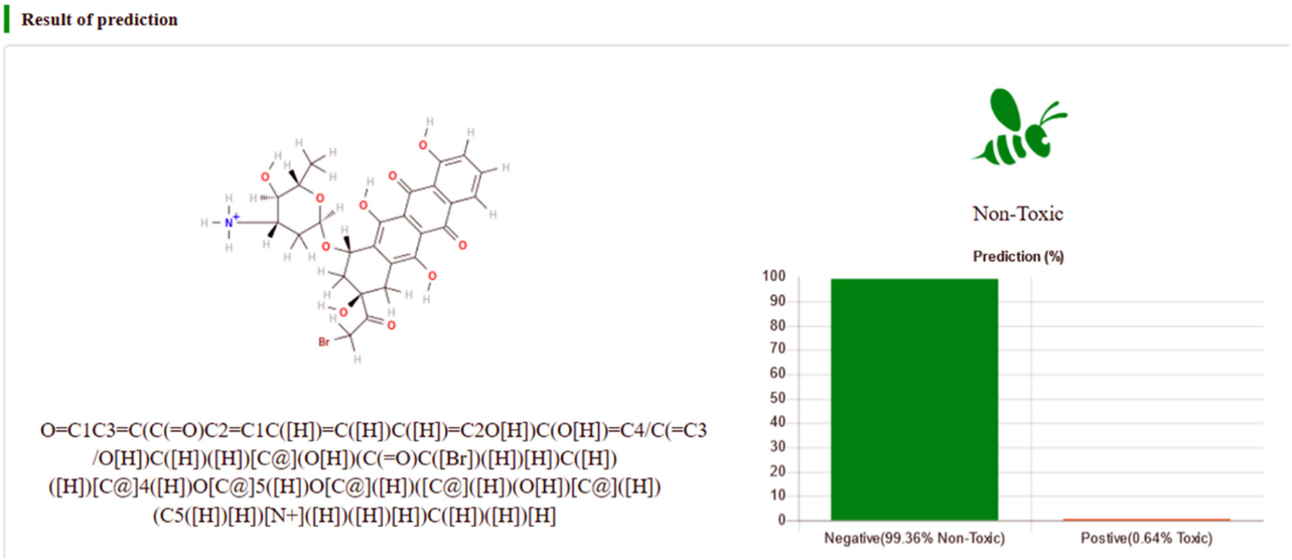

Figure S5. AH-632/20791006 (Compound 2 from Table 2).

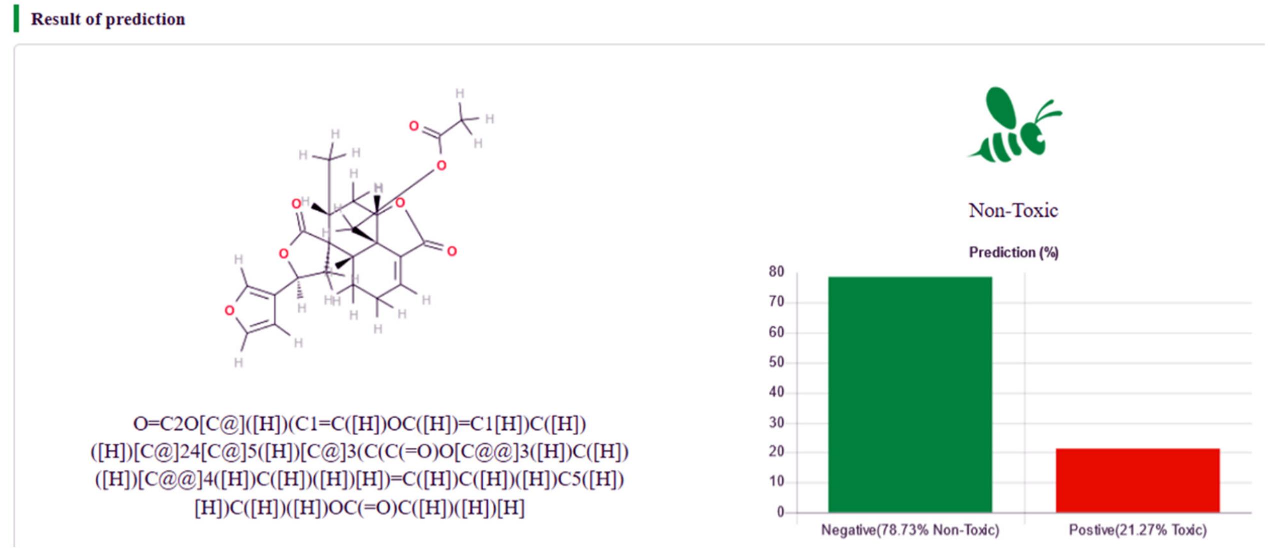

Figure S6. AO-166/21204006 (Compound 4 from Table 2).

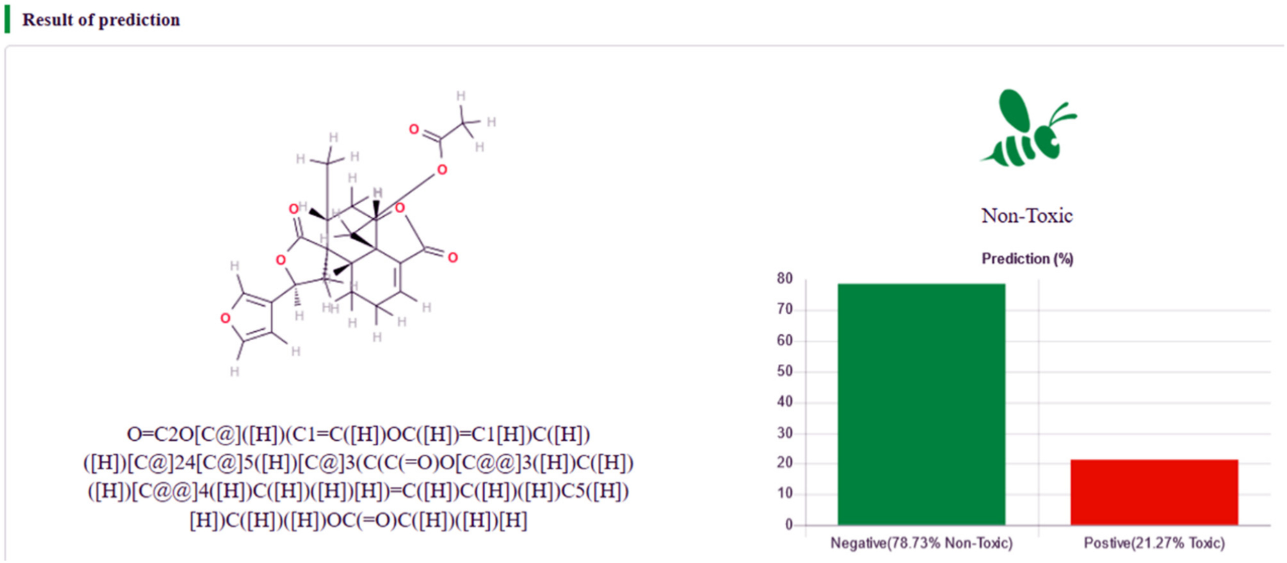

Figure S7. AO-222/41148840 (Compound 5 from Table 2).

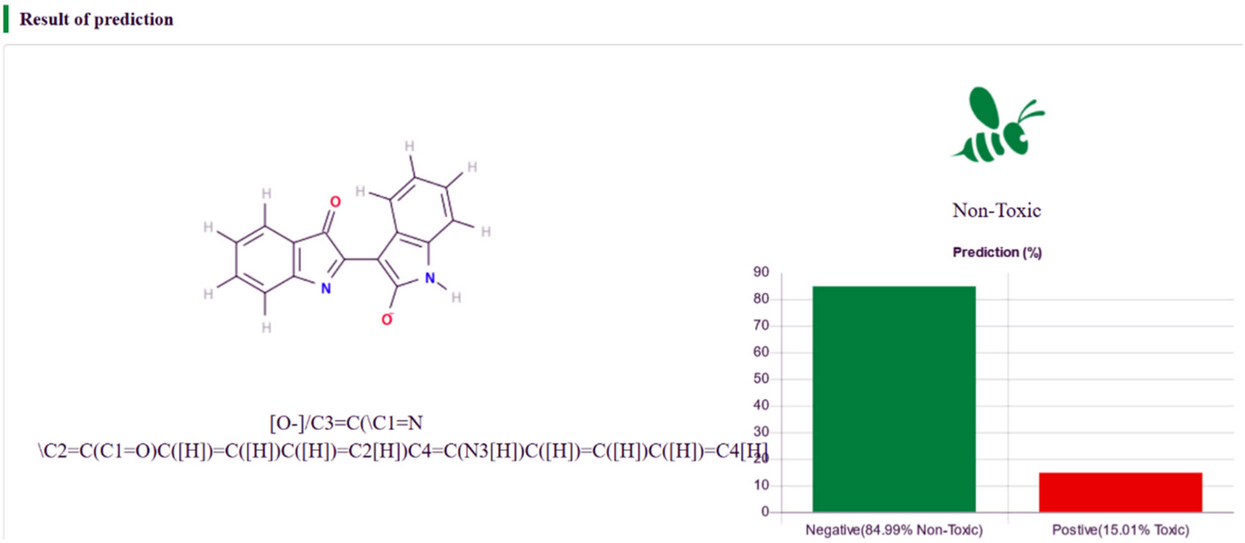

Figure S8. AM-331/20711002 (Compound 6 from Table 2).

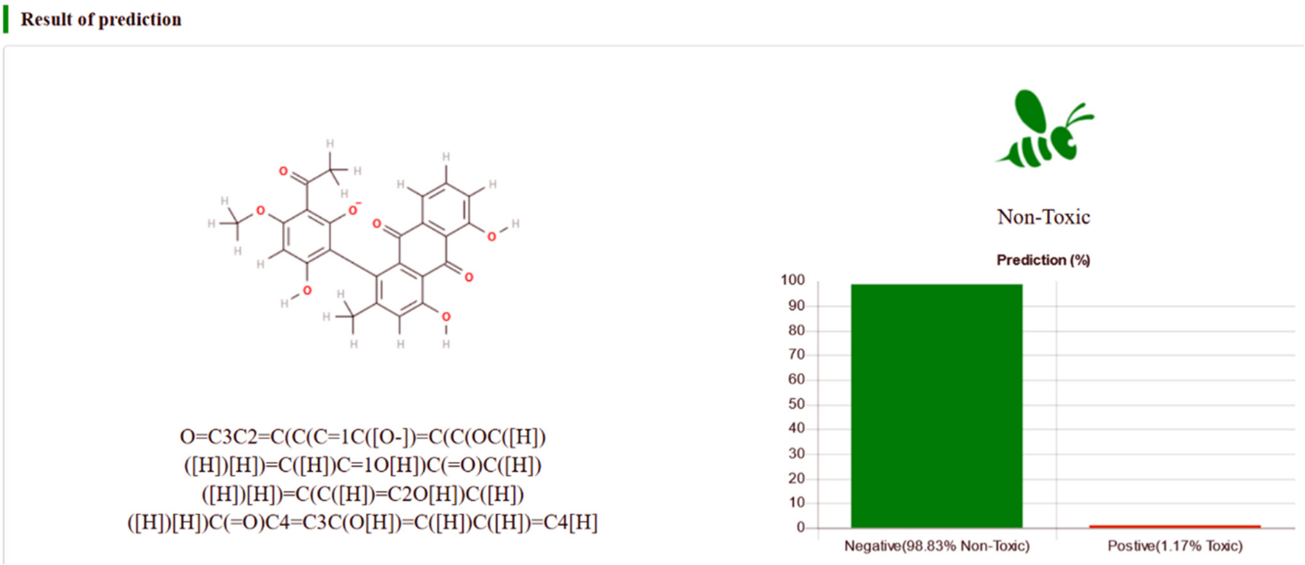

Figure S9. AI-899/21033027 (Compound 7 from Table 2).

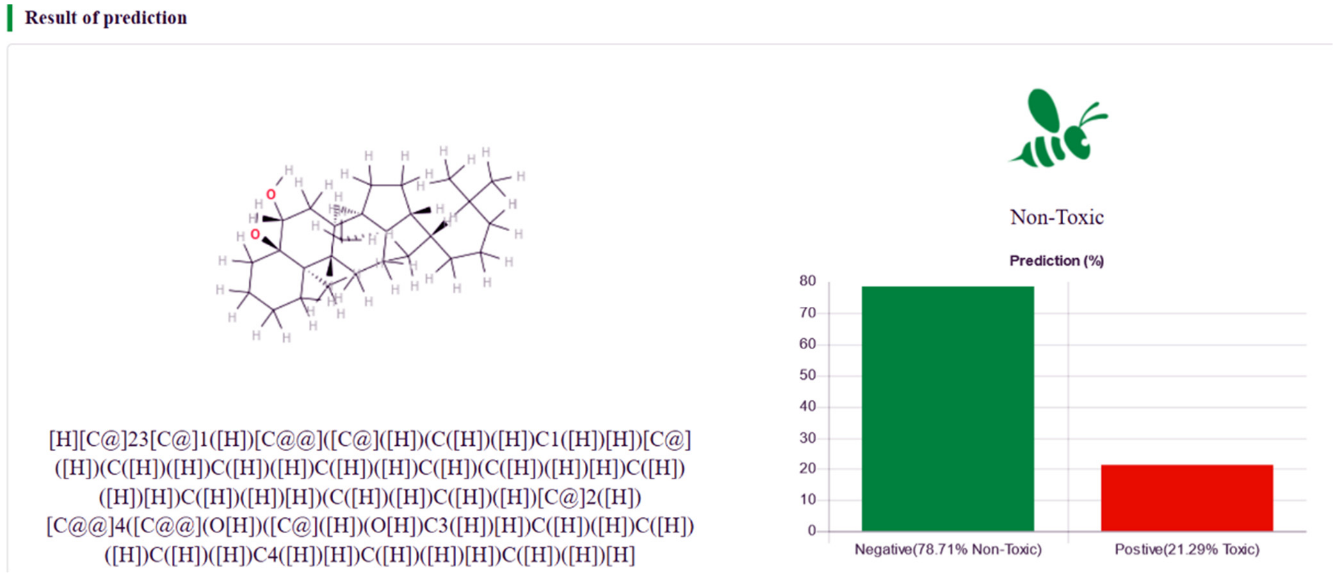

Figure S10. AQ-152/40869673 (Compound 8 from Table 2).

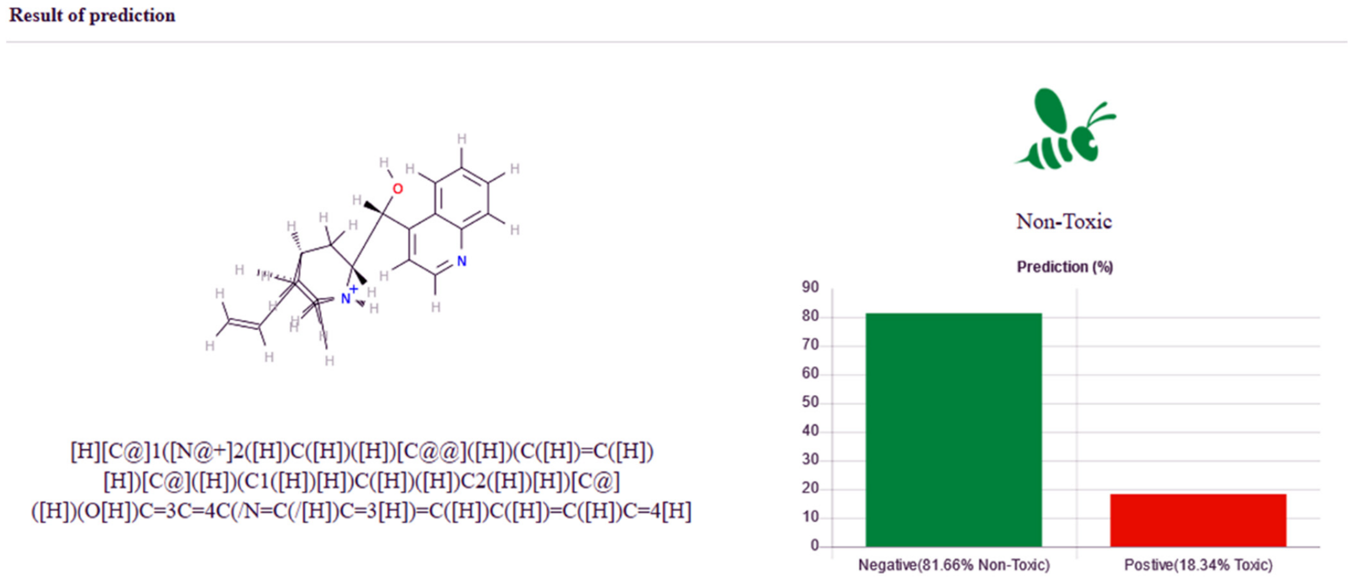

Figure S11. AL-466/21162039 (Compound 10 from Table 2).

Table S5. Bioconcentration Factors (BCFs) computed with BCFBAF Program from EPI Suite.

Crt No	Specs ID number	BCF (L/kg wet-wt)
1	AO-253/40760091	3.16
2	AH-632/20791006	3.16
3	AE-508/21132035	2.58e+003
4	AO-166/21204006	3.37e+003
5	AO-222/41148840	11.3
6	AM-331/20711002	40.5
7	AI-899/21033027	140
8	AQ-152/40869673	5.85e+003
9	AO-774/41465391	385
10	AL-466/21162039	3.16

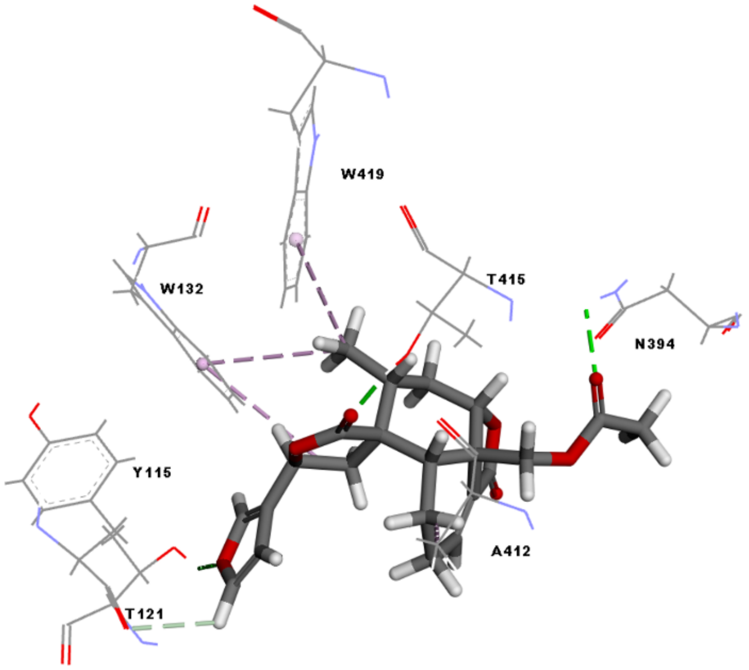

Figure S12. The compound 5 into the DAR AgDOP2 binding site. The hydrophilic interactions are depicted with green dashed lines, while hydrophobic interactions are presented with pink dashed lines.
